# COVID-19 and Mortality in the Spinal Cord Injury Population: Examining the Impact of Sex, Mental Health, and Injury Etiology

**DOI:** 10.3390/healthcare12192002

**Published:** 2024-10-07

**Authors:** Arrani Senthinathan, Mina Tadrous, Swaleh Hussain, Aleena Ahmad, Cherry Chu, B. Catharine Craven, Susan B. Jaglal, Rahim Moineddin, Lauren Cadel, Vanessa K. Noonan, John Shepherd, Sandra McKay, Karen Tu, Sara J. T. Guilcher

**Affiliations:** 1 Leslie Dan Faculty of Pharmacy, University of Toronto, Toronto, ON M5S3H2, Canada; 2 Holzschuh College of Business Administration, Niagara University, Lewiston, NY 14109, USA; 3 ICES, Toronto, ON M4N 3M5, Canadasara.guilcher@utoronto.ca (S.J.T.G.); 4 Women’s College Hospital Institute for Health Systems Solutions and Virtual Care, Women’s College Hospital, Toronto, ON M5S1B2, Canada; 5 Department of Biology, University of Toronto Mississauga, Mississauga, ON M5S1B2, Canada; 6 Institute of Health Policy Management and Evaluation, University of Toronto, Toronto, ON M5S1B2, Canada; 7 KITE (Knowledge Innovation Talent Everywhere), Toronto Rehabilitation Institute, University Health Network, Toronto, ON M5G2A2, Canada; 8 Department of Medicine, Faculty of Medicine, University of Toronto, Toronto, ON M5S1A8, Canada; 9 Spinal Cord Rehabilitation Program, Toronto Rehabilitation Institute, University Health Network, Toronto, ON M5G2A2, Canada; 10 Department of Physical Therapy, Temerty Faculty of Medicine, University of Toronto, Toronto, ON M5S1A8, Canada; 11 Rehabilitation Science Institute, University of Toronto, Toronto, ON M5R0A3, Canada; 12 Department of Family and Community Medicine, University of Toronto, Toronto, ON M5G1V7, Canada; 13 International Collaboration on Repair Discoveries, Vancouver, BC V5Z1M9, Canada; 14 Praxis Spinal Cord Institute, Vancouver, BC V5Z1M9, Canada; 15 VHA Home HealthCare, Toronto, ON M4S1V6, Canada; 16 North York General Hospital, Toronto, ON M2K1E1, Canada; 17 Toronto Western Family Health Team, University Health Network, Toronto, ON M5T2S6, Canada

**Keywords:** COVID-19, pandemic, mortality, spinal cord injury

## Abstract

Background/Objective: The purpose of this study was to investigate the impact of the COVID-19 pandemic on mortality rates in a community-dwelling spinal cord injury (SCI) population in Ontario. Methods: Using health administrative databases, monthly mortality rates were evaluated pre-pandemic, during the pandemic, and post-pandemic from March 2014 to May 2024. Data were stratified by sex, injury etiology, and mental health status. Group differences were evaluated using *t*-tests. Autoregressive integrated moving average (ARIMA) models evaluated the pandemic’s impact on mortality rates. Results: A significant increase of 21.4% in mortality rates during the pandemic was found for the SCI cohort. With the exception of the traumatic group, all subgroups also experienced a significant increase in mortality rates (males: 13.9%, females: 31.9%, non-traumatic: 32.3%, mental health diagnoses: 19.6%, and mental health diagnoses: 29.4%). During the pandemic, females had a significantly higher mortality rate than males. The non-traumatic group had higher mortality rates than the traumatic group at all time periods. Individuals with mental health diagnoses had higher mortality rates than those without at the pre-pandemic and pandemic periods. Conclusions: The variation in mortality rates across groups highlights inequitable access to medical care in the SCI population, with further research and interventions needed.

## 1. Introduction

The COVID-19 pandemic has resulted in unprecedented global mortality rates [1]. As of mid-2024, the World Health Organization (WHO) estimates over 7 million deaths can be directly attributed to the COVID-19 virus [1]. Beyond direct viral infections, the pandemic had a greater indirect impact on mortality rates, with over 18 million excess deaths estimated globally between January 2020 and December 2021 [2]. Excess deaths account for both the total number of deaths directly attributed to the virus, as well as indirect impacts. Reasons for indirect impacts include shifts in healthcare access and delivery, as well as social determinants of health [2,3]. A systematic review and meta-analysis found in a regional analysis that North America had the second highest region excess mortality (134.02 deaths per 100,000) globally [4]. Males (130.10 per 100,000) and those over 60 years old (781.74 per 100,000) exhibited higher mortality rates during the pandemic [4]. Research has not investigated the combined multimorbidity associated with disability, medical comorbidities, and mental health factors on mortality rates among individuals living in the community. 

The pandemic has highlighted healthcare inequities among marginalized and vulnerable populations, including individuals with disabilities [5,6,7,8]. A previous study conducted from January 2020 to January 2021 in Ontario, Canada reported that adults with intellectual and developmental disabilities were twice as likely to die following a COVID-19 infection compared to adults without intellectual and developmental disabilities [5]. Further, a separate study evaluating 1279 COVID-19 hospital admissions in Ontario from January 2020 to November 2020 found, in an unadjusted model, that patients with a disability were significantly more likely to die than those without a disability when infected with COVID-19.

Among persons with spinal cord injury (SCI), higher mortality rates have been identified among those infected with COVID-19 [9]. A study from the United States using the Veterans Health Administration (VHA) National Spinal Cord Injury and Disorders Registry from 15 January 2020 to 10 January 2021 found that individuals with SCI and a positive COVID-19 test had a mortality rate of 12%, which is higher than the national average of less than 1% [9]. Individuals with SCI may be at higher risk of severe outcomes from COVID-19 due to compromised respiratory function [10]. Individuals with SCI often have weakened or paralyzed chest muscles, reducing their ability to cough effectively and clear secretions from the lungs, which increases their susceptibility to respiratory infections [10]. As such, the COVID-19 infection most likely would further diminish their ability to cope with inflammation and fluid buildup, making recovery more difficult and increasing the likelihood of fatal outcomes [10,11].

Beyond the COVID-19 virus, individuals with SCI who are dependent on others for care likely faced unique challenges during the pandemic in accessing healthcare, maintaining social distancing, and adhering to preventive measures [6,12]. A previous scoping review investigating the impact of the pandemic on individuals with SCI found, in multiple countries and healthcare systems, that those residing in the community experienced increases in secondary health conditions and worse health outcomes, decreases in healthcare utilization and access, and shifts in formal and informal caregiving support [6]. As such, these factors and others may have also indirectly impacted rates of mortality in the SCI population beyond the COVID-19 viral infection. 

To date, no North American studies have investigated overall shifts in mortality rates in the SCI population or other disability populations to evaluate excess deaths due to both the direct and indirect effects of the pandemic. Understanding the impact of the COVID-19 pandemic on mortality rates within the SCI population is crucial for developing targeted interventions and support systems. The purpose of this study was to investigate the impact of the COVID-19 pandemic on mortality rates in an SCI population currently living in the community.

## 2. Methods

This was a cohort study using linked health administrative databases from the ICES in Ontario, Canada’s largest province by population with over 14 million people [13]. Monthly mortality rates were evaluated from March 2014 to May 2024. The pre-pandemic period was defined as March 2014 to February 2020, the pandemic period as March 2020 to April 2023, and the post-pandemic period as May 2023 to May 2024 [14]. 

### 2.1. Data Source 

Data for this study were obtained from the ICES, an independent, non-profit research institute funded by an annual grant from the Ontario Ministry of Health. The data used from this study were obtained from the ICES, which is an institute that provides secure access to Ontario’s health administrative data. As a prescribed entity under Ontario’s privacy legislation, the ICES is authorized to collect and use healthcare data for the purposes of health system analysis, evaluation, and decision support. Secure access to these data is governed by policies and procedures that are approved by the Information and Privacy Commissioner of Ontario.

### 2.2. Cohorts

The SCI cohort for this study comprised individuals with traumatic and non-traumatic injuries. Individuals with traumatic SCI (TSCI) were identified from the Discharge Abstract Database (DAD) based on first admission and discharge from acute hospitalization between 1 April 2004, and 28 February 2014. Individuals with non-traumatic SCI (NTSCI) were identified from the National Rehabilitation System (NRS) database based on first admission and discharge from all facilities with designated adult inpatient rehabilitation beds between 1 April 2004, and 28 February 2014. Individuals were excluded from the cohort if they died before the end of their index episode of care, if they were not previously discharged from acute hospitalization or inpatient rehabilitation before 2004, or if they were not eligible for public health insurance coverage through the Ontario Health Insurance Plan (OHIP). Individuals were also excluded if they had a missing date of birth or sex variable. 

### 2.3. Variables 

The main outcome measure of interest was monthly mortality rates obtained from the Registered Persons Database (RPDB) based on death date. Other patient characteristics collected included socio-demographics (age, sex, and postal code). Postal codes were used to identify rurality and neighbourhood income. 

A history of mental health diagnosis/es was identified in the DAD and OHIP databases based on flags in the multimorbidity macro for mood disorders and other mental health conditions [15]. The presence of either or both conditions indicated that an individual had a history of mental health conditions or a mental health diagnosis. The multimorbidity macro was also used to calculate the number of health conditions. The number of medications was obtained from the Ontario Drug Benefits Database (ODB). The study had a two-year lookback period to identify comorbidity information prior to 1 March 2020, and a one year look back to identify prescription drugs prior to 1 March 2020.

The Johns Hopkins ACG^®^ System Version 10 was used to categorize individuals by Resource Utilization Bands (RUBs). RUBs are indicators of expected health-related resource utilization and vary from 0 to 5, with higher values associated with higher utilization levels. For this study, proprietary software determined the number of RUBs with ACG^®^ System Aggregated Diagnosis Groups (ADGs) using hospitalization, emergency department (ED) visits, and physician office visits based on diagnosis data from ICES databases.

### 2.4. Data Analysis 

Descriptive statistics were conducted to summarize demographic, clinical, and injury characteristics. Continuous variables were expressed as means and standard deviations, and categorical variables were expressed as frequencies and percentages. Monthly mortality rates were calculated from March 2014 to October 2023. The mortality rate was expressed as the number of deaths per 1000 individuals. Data were stratified by sex (male or female), injury etiology (TSCI or NTSCI), and mental health status (yes or no) subgroups. Groups were identified based on their status at the start of the observation period (March 2014). *t*-tests were conducted to evaluate between-group differences during the pre-pandemic, pandemic, and post-pandemic periods. 

To evaluate the impact of the COVID-19 pandemic on mortality rates for the full cohort and within subgroups, a time-series analysis was conducted using interventional autoregressive integrated moving average (ARIMA) modelling. Interventional ARIMA is a common technique used to better understand the impact of a significant event or policy change on time series data while accounting for important components related to trend, seasonality, and autocorrelation [16]. Two ramp functions were incorporated into the models representing the pandemic period (March 2020 to April 2023) and the post-pandemic period (May 2023 to May 2024), respectively. A *p*-value of less than 0.05 indicates a significant shift in mortality rates during those time periods. The model was differenced in cases where the time series was not stationary (as indicated by the augmented Dickey–Fuller test) and seasonal differencing was applied if there was any seasonality. The optimal number of autoregressive or moving average terms was determined using autocorrelation function (ACF) and partial autocorrelation function (PACF) plots, Akaike information criterion and Schwarz–Bayesian criterion indicators, and Ljung–Box χ^2^ tests for white noise. 

## 3. Results

At the start of our observation period in February 2014, 5753 individuals with SCI were included in our study. At the pandemic onset in March 2020, 4405 individuals were still alive, and at the end of the pandemic in May 2023, 3715 individuals were alive. For full cohort and demographic details, please see Table 1. 

### 3.1. Group Differences for Mortality Rates 

No significant differences were found between males and females pre-pandemic and post-pandemic in monthly mortality rates. During the pandemic, females had a significantly higher monthly mortality rate than males (t (74) = 2.56, *p* = 0.012). The NTSCI group had higher monthly mortality rates than the TSCI group during the pre-pandemic (t (142) = 9.35, *p* < 0.0001), pandemic (t (74) = 12.00, *p* < 0.0001), and post-pandemic (t (24) = 4.32, *p* = 0.0002) periods. Those with mental health diagnoses had higher monthly mortality rates than those without during the pre-pandemic (t (142) = 4.62, *p* < 0.0001) and pandemic periods (t (74) = 2.78, *p* = 0.0068) (Table 2). 

### 3.2. ARIMA Results

**Figure 1:**
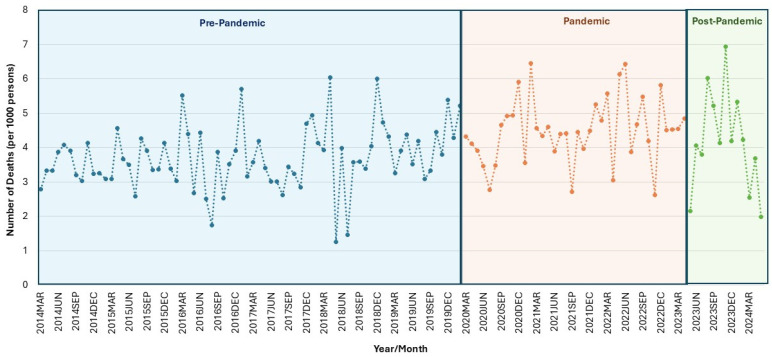
Mortality rate for spinal cord injury population in Ontario, Canada. Monthly number of deaths per 1000 persons for full spinal cord injury cohort across pre-pandemic, pandemic, and post-pandemic periods.

**Figure 2:**
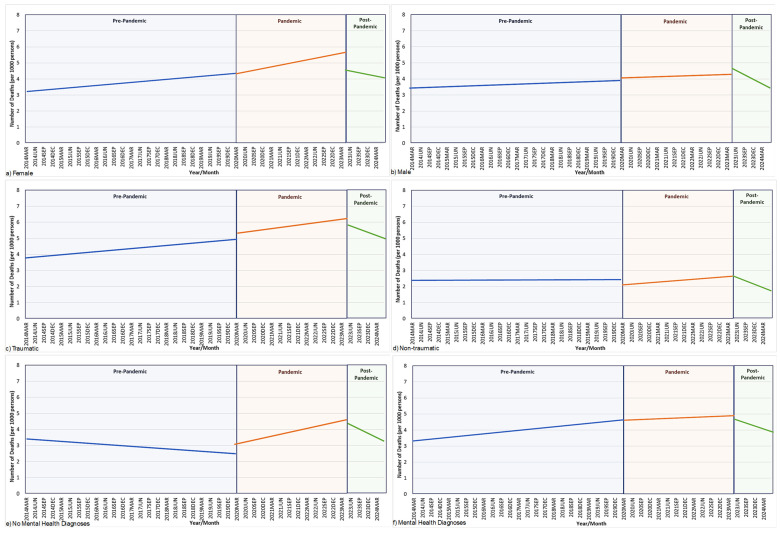
Mortality rate for spinal cord injury population stratified by sex, injury etiology, and mental health status. Number of monthly deaths per 1000 persons for spinal cord injury population across the pre-pandemic, pandemic, and post-pandemic periods for (**a**) females, (**b**) males, (**c**) traumatic spinal cord injury, (**d**) non-traumatic spinal cord injury, (**e**) no mental health diagnosis, and (**f**) mental health diagnosis. To avoid the reporting or re-calculation of small cells, only trend lines were reported in figures.

For the full cohort, a significant increase in mortality rates of 21.4% was found from the pre-pandemic period to the pandemic period (*p* < 0.0001) (Figure 1, Table 3). Significant increases in mortality rates were also found for males (13.9%), females (31.9%), individuals with NTSCI (32.3%), and those with mental health diagnoses (19.6%) and without mental health diagnoses (29.4%) during the pandemic compared to the pre-pandemic period (Figure 2, Table 3). No significant changes from the pre-pandemic to pandemic period were found in the TSCI group. Only males experienced a decrease in mortality rates post-pandemic compared to the pandemic period (*p* = 0.0462). 

## 4. Discussion

We found that the community-dwelling SCI population in Ontario experienced an increase of 21.4% in mortality rates during the pandemic. Unfortunately, these results are not surprising given the risk factors among persons with SCI, including a high number of comorbidities, lower socioeconomic status, and high healthcare needs [6,12,17,18] in a region with high COVID-19 prevalence [1]. It should be noted that the increase in deaths found in this study is due to a combination of direct deaths caused by the COVID-19 infection and indirect factors due to the COVID-19 pandemic. Females had a significantly higher mortality rate compared to males during the pandemic but not during the pre-pandemic or post-pandemic periods. This may suggest that females were more impacted by the pandemic. Pre-pandemic differences existed between injury etiology and mental health status subgroups, and the pandemic did not seem to exacerbate these differences. Apart from the TSCI group, all other subgroups experienced a significant increase in their mortality rates during the pandemic. 

When considering mortality rates, it is important to consider other outcomes and factors that may have contributed to the observed rates, such as pre-pandemic health status, healthcare needs, utilization, and delivery patterns. A previous study conducted in Ontario investigating healthcare utilization patterns during the pandemic in the same community-dwelling SCI cohort found differences in the TSCI and NTSCI population [12]. The study found that, compared to pre-pandemic levels, both TSCI and NTSCI groups experienced a significant decrease in their emergency department visits (*p* = 0.0257 for TSCI and *p* = 0.0016 for NTSCI), but only NTSCI experienced a significant reduction in hospital admissions (*p* =  0.0088) at the pandemic onset [12]. This study also indicated individuals with TSCI and NTSCI experienced an initial decrease in physician visits with the pandemic onset; however, the rapid increase in their virtual visits compensated for the decrease in their in-person healthcare [12]. A scoping review evaluating the healthcare experiences of individuals with SCI during the pandemic globally found a high level of satisfaction with using virtual care platforms, and high uptake of virtual care [6]. The approval and rapid uptake of virtual care [6,12] as well as decreased levels of emergency department visits and hospital admissions [12] may suggest adequate healthcare access for the SCI population during the pandemic. Conversely, our finding of increased rates in mortality suggests individuals may not have been recognized as having COVID-19 and/or may not have received the care they needed during the pandemic. The differences in hospital admission rates between TSCI and NTSCI may also be a factor in differences in mortality rates between the two groups during the pandemic. Shifts in emergency department visits and hospital admission rates may be due to a reluctance to access care due to a fear of exposure to the COVID-19 virus [6,12].

During the pandemic, the SCI population also experienced shifts in formal and informal caregiving support [6,18]. The scoping review evaluating the healthcare experiences of individuals with SCI reported that individuals with SCI had difficulty maintaining formal or paid caregiving services and relied more heavily on informal caregiving from family [6]. A study investigating professional homecare services for individuals with SCI in Ontario during the pandemic found an increase in nursing services for both individuals with TSCI and NTSCI, but only those with TSCI experienced a decrease in personal support services [18]. This may indicate that individuals with NTSCI had higher healthcare needs during the pandemic, explaining the increase in mortality rates in this population during the pandemic. However, maintaining personal support services during the pandemic may have increased the risk of infection in the NTSCI group, which may have contributed to increased rates of mortality as well. These shifts in healthcare utilization, care delivery, and caregiving support may have contributed to the observed increase in mortality in the SCI population, as well as explain the differences in mortality rate between the TSCI and NTSCI groups due to comorbid conditions. 

We found that both female and male individuals with SCI experienced an increase in mortality rates, with females experiencing significantly higher rates during the pandemic. These findings differ from previous research, which found that males in the general population were more likely to experience worse health outcomes if infected with the COVID-19 virus, including an increased risk of mortality [19,20,21]. There are biological differences, with males having a weaker immune response to COVID-19 infections [20,21]. Studies have found that males also have higher rates of comorbidities, which leads to more severe outcomes when infected with COVID-19 [19]. There are also lifestyle and behavioural factors, with males exhibiting higher rates of smoking, delayed healthcare seeking, and decreased hand hygiene practices, which may also contribute to increased mortality risk among males when infected with COVID-19 [19,20,21]. However, our community-dwelling SCI population may experience alternative factors, which may have contributed to the increased mortality rates in females. For example, the females in our cohort may have had higher healthcare needs and comorbidities compared to the general population. They may have also experienced issues with accessing care or adequate caregiving support [6,12,18]. The findings from our study outline shifts in care and different needs in the SCI population across sexes when compared to the general population. Future research should continue to investigate sex and gender differences in the SCI population with regards to healthcare access, delivery, and outcomes, as well as caregiving support, during the pandemic and afterwards. 

Individuals with SCI, both with and without concurrent mental health diagnoses, experienced an increase in mortality rates during the pandemic compared to the pre-pandemic period. However, those with mental health diagnoses had higher rates of mortality pre-pandemic (3.97 deaths per 1000 persons vs. 2.96 deaths per 1000 persons) and during the pandemic (4.75 deaths per 1000 persons vs. 3.83 deaths per 1000 persons). A scoping review evaluating the impact of the pandemic on those with pre-existing mental health conditions found that individuals with mental health conditions in the general population experienced social isolation, loneliness, and reduced access to health services and treatments [22]. This can lead to an exacerbation of their conditions, putting them at a greater risk of COVID-19 infection and severe outcomes, including mortality. A systematic review and meta-analysis evaluating the impact of mental disorders on COVID-19 mortality and related outcomes supported this conclusion, as it found an increased risk of hospitalization (OR: 2.24, 95% CI: 1.70–2.94) and COVID-19 mortality (OR: 2.00, 95% CI: 1.58–2.54) during the pandemic for those with mental health conditions [23]. Our study found no differences in post-pandemic mortality rates between those with and without mental health diagnoses. However, groups were determined based on status at the start of the observation period in February 2014. It is possible that those without mental conditions at the start of the observation period may have developed them as time progressed. As such, this may explain the lack of difference between the two groups during the post-pandemic period. 

Another key component to consider when evaluating mortality during the pandemic is COVID-19 vaccine uptake. During the pandemic, Ontario prioritized the vaccination of vulnerable populations [24,25]. As such, individuals with SCI were often categorized as a priority group for the vaccine rollout [26]. However, it is unknown if COVID-19 vaccine uptake by the SCI population aligns with recommendations. It is important for future research to evaluate differences in vaccine uptake in the SCI subgroups as it may provide insight into differences in pandemic mortality rates between sexes, mental health status, and injury etiology.

### Limitations

This study comprised the secondary use of health administrative data and hence was limited by the extent and type of data available in the databases. We did not evaluate the reasons for death. We chose to focus on the overall rates of morality, with the goal of determining excess deaths due to the pandemic. We were unable to obtain information on severity and level of injury for both the NTSCI and TSCI group; hence, future studies should investigate this factor. This study focused on a fixed cohort of a community-dwelling SCI population in Ontario. Furthermore, population subgroups (sex, injury etiology, and mental health status) were identified in February 2014. As such, the generalizability of results to other health systems and populations should be carried out with caution. The majority of our cohort resides in an urban area; hence, the generalizability of our findings to those who live in rural areas is limited, as individuals in rural areas may face greater or different barriers to healthcare access. We conducted multiple *t*-tests without correction, increasing the risk of a Type I error. However, we did not apply corrections for multiple comparisons as our intention was to identify potential differences between groups that could warrant further investigation for future studies.

## 5. Conclusions

Our study found that the COVID-19 pandemic significantly increased mortality rates in the community SCI cohort. We also found varying mortality rates during the pre-pandemic, pandemic, and post-pandemic periods in SCI subgroups. This highlights the importance of continued investigation into the influence of sex, injury etiology, and mental health status within the SCI population. We also found that females, individuals with NTSCI, and those with mental health diagnoses experienced increased mortality rates during different phases of the observation. As such, further research is needed to explore the specific factors and interventions that contributed to these outcomes to ensure equitable access to care and healthcare resources. Understanding these dynamics is crucial for developing targeted strategies and policies that can improve health outcomes and support for vulnerable populations within the SCI community.

## Figures and Tables

**Table 1:** Spinal cord injury cohort demographic and clinical information at the start of the pre-pandemic, pandemic, and post-pandemic periods.

	Pre-Pandemic (February 2014) N = 5753		Pandemic (March 2020) N = 4405		Post-Pandemic (May 2023) N = 3715	
Characteristics						
	n	%	n	%	n	%
Sex						
Female	2353	40.9	1795	40.7	1488	40.1
Male	3400	59.1	2610	59.3	2227	59.9
Age (Years)						
Mean (Standard Deviation)	60.7 (18.4)		62.85 (18.0)		63.43 (17.6)	
Median (Quintile 1–3)	63 (49–75)		65 (51–77)		65 (51–77)	
Injury Etiology						
Non-Traumatic	3868	67.2	2824	64.1	2270	61.1
Traumatic	1885	32.8	1581	35.9	1445	38.9
Time Since Injury (Years)						
Mean (Standard Deviation)	4.1 (2.8)		10.1 (2.8)		10.1 (2.8)	
Median (Quintile 1–3)	4 (2–6)		10 (8–12)		10 (8–12)	
Rurality						
Rural	474	8.2	387	8.8	343	9.2
Urban	5197	90.3	3947	89.6	3302	88.9
Income Quintile						
Quintile 1 (low)	1393	24.2	1067	24.2	930	25.0
Quintile 2	1218	21.2	1002	22.8	806	21.7
Quintile 3	1134	19.7	807	18.3	688	18.5
Quintile 4	951	16.5	737	16.7	612	16.5
Quintile 5 (high)	1038	18.0	767	17.4	656	17.7
Resource Utilization Bands (RUBs)						
0 to 1 (low)	141	2.4	307	7.0	347	9.3
2 to 3	1892	32.9	1908	43.3	1618	43.6
4 to 5 (high)	3720	64.7	2190	49.7	1750	47.1
Mental Health Diagnosis						
Yes	1859	32.3	1218	27.7	1005	27.1
No	3894	67.7	3187	72.3	2710	72.9
Number of Health Conditions						
0 to 2	1632	28.4	1086	24.7	948	25.5
3 to 4	1835	31.9	1376	31.2	1155	31.1
5+	2286	39.7	1943	44.1	1612	43.4

**Table 2:** Average monthly mortality rates per 1000 persons pre-pandemic, pandemic, and post-pandemic for spinal cord injury population across full cohort, sex, injury etiology, and mental health status.

Cohort	Pre-Pandemic (March 2014–February 2020)			Pandemic (March 2020–April 2023)			Post-Pandemic (May 2023–May 2024)		
	Average	Standard Deviation	*t*-Test Results	Average	Standard Deviation	*t*-Test Results	Average	Standard Deviation	*t*-Test Results
All	3.70	0.92		4.49	0.96		4.18	1.46	
Male	3.66	1.11	t (142) = 0.46, *p* = 0.64	4.17	1.12	t (74) = 2.56, *p* = 0.012	4.09	1.97	t (24) = 0.28, *p* = 0.78
Female	3.76	1.48		4.96	1.53		4.32	2.20	
Traumatic	2.44	1.17	t (142) = 9.35, *p* < 0.0001	2.35	1.11	t (74) = 12.00, *p* < 0.0001	2.22	1.54	t (24) = 4.32, *p* = 0.0002
Non-Traumatic	4.36	1.29		5.77	1.36		5.46	2.22	
Mental Health Diagnosis	3.97	1.14	t (142) = 4.62, *p* < *0*.0001	4.75	1.18	t (74) = 2.78, *p* = 0.0068	4.31	1.62	t (24) = 0.52, *p* = 0.61
No Mental Health Diagnosis	2.96	1.46		3.83	1.66		3.85	2.74	

**Table 3:** ARIMA model results comparing mortality rates pre-pandemic, pandemic, and post-pandemic for spinal cord injury population across full cohort, sex, injury etiology, and mental health status.

Cohort	Pandemic Compared to Pre-Pandemic Mortality Rates		Post-Pandemic Compared to Pandemic Mortality Rates	
	% Change	ARIMA (*p*-Value)	% Change	ARIMA (*p*-Value)
All	21.4	<0.0001	−6.9	0.2787
Male	13.9	<0.0001	−1.9	0.0462
Female	31.9	0.0002	−12.9	0.5158
Traumatic	−3.7	0.9119	−5.5	0.3771
Non-Traumatic	32.3	0.0001	−5.4	0.3219
Mental Health Diagnosis	19.6	0.0009	−9.3	0.9752
No Mental Health Diagnosis	29.4	0.0136	0.5	0.386

## Data Availability

The dataset from this study is held securely in coded form at the ICES. While legal data sharing agreements between the ICES and data providers (e.g., healthcare organizations and government) prohibit the ICES from making the dataset publicly available, access may be granted to those who meet pre-specified criteria for confidential access, available at www.ices.on.ca/DAS (email: das@ices.on.ca) (accessed on 1 October 2024). The full dataset creation plan and underlying analytic code are available from the authors upon request, understanding that the computer programs may rely upon coding templates or macros that are unique to the ICES and are therefore either inaccessible or may require modification.
